# Effect of gestational diabetes mellitus on lipid profile: A systematic review and meta-analysis

**DOI:** 10.1515/med-2021-0408

**Published:** 2021-12-15

**Authors:** Fatemeh Alsadat Rahnemaei, Reza Pakzad, Azam Amirian, Iraj Pakzad, Fatemeh Abdi

**Affiliations:** Reproductive Health Research Center, Department of Obstetrics & Gynecology, Al-zahra Hospital, School of Medicine, Guilan University of Medical Sciences, Rasht, Iran; Department of Epidemiology, Faculty of Health, Ilam University of Medical Sciences, Ilam, Iran; Department of Midwifery, School of Nursing and Midwifery, Jiroft University of Medical Sciences, Jiroft, Iran; School of Allied Medical Sciences, Ilam University of Medical Sciences, Ilam, Iran; Cardiovascular Research Center, Alborz University of Medical Sciences, Karaj, Iran; Non-communicable Diseases Research Center, Alborz University of Medical Sciences, Karaj, Iran

**Keywords:** gestational diabetes mellitus, lipid profile, triglyceride, TG/HDL ratio, total cholesterol, LDL-C, HDL-C, VLDL-C

## Abstract

Gestational diabetes mellitus (GDM) can have adverse effects on pregnancy. GDM is associated with changes in the lipid profile of pregnant women. Finding out the early ways to diagnose GDM can prevent the adverse outcomes. This meta-analysis study aimed to determine the effect of GDM on lipid profile. PubMed, ProQuest, Web of Science, Scopus, Science Direct, Google Scholar, and ClinicalTrial were systematically searched for published articles relating to GDM until 2021 according to PRISMA guidelines. Newcastle Ottawa scale was used to assess the quality of the studies. Thirty-three studies with a sample size of 23,792 met the criteria for entering the meta-analysis. Pooled standardized mean difference (SMD) for total cholesterol (TC) and triglyceride (TG) was 0.23 mg/dL (95% CI: 0.11–0.34) and 1.14 mg/dL (95% CI: 0.91–1.38), respectively. The mean of TC and TG in people with GDM was higher than that in normal pregnant women. A similar pattern was observed for the very low-density lipoprotein (VLDL) and TG/high-density lipoprotein (HDL) ratio, with pooled SMD of 0.99 mg (95% CI: 0.71–1.27) and 0.65 mg (95% CI: 0.36–0.94), respectively. Pooled SMD for HDL was −0.35 mg/dL (95% CI: −0.54 to −0.16), women with GDM had a mean HDL lower than normal pregnant women. Although pooled SMD was higher for low-density lipoprotein (LDL) in the GDM group, this difference was not significant (0.14 [95% CI: −0.04 to 0.32]). Of all the lipid profiles, the largest difference between the GDM and control groups was observed in TG (SMD: 1.14). Elevated serum TG had the strongest effect on GDM. Higher levels of TC, LDL, VLDL, and TG/HDL ratio, and lower level of HDL were exhibited in GDM group. So, these markers can be considered as a reliable marker in the diagnosis of GDM.

## 1 Introduction

Gestational diabetes mellitus (GDM) is the most common metabolic disorder during pregnancy and is defined as diabetes identified in the second or third trimester of pregnancy that was not previously known. A possible cause of GDM is an exacerbation of physiological changes in glucose metabolism during pregnancy [1]. Pregnancy as a complex process leads to physiological changes in the female body. Most pregnant women go through pregnancy safely; however, some of them develop complications such as gestational diabetes. Myo-inositol and d-chiro-inositol are natural compounds involved in many biological pathways and both are currently well tolerated. They are effective alternatives to classical insulin sensitizers and are useful in the prevention and treatment of metabolic and reproductive disorders such as polycystic ovary syndrome and GDM [2,3]. In the last decade, the prevalence of GDM has increased due to inactivity, obesity, and increasing age of mothers. One in ten pregnancies is diagnosed with diabetes, 90% of which is identified as GDM. The prevalence of GDM is estimated at 17% worldwide. It is reported to be 10% in North America and 25% in Southeast Asia, depending on population, region, diagnostic criteria, and methods of data collection [4]. According to the World Health Organization (WHO), diabetes is reported as the seventh cause of human death [5]. GDM is considered as a silent disease that can have adverse effects on the mother and fetus and lead to undesirable consequences such as polyhydramnios, pre-eclampsia, stillbirth, fetal macrosomia, hyperbilirubinemia, hypocalcemia, hypoglycemia, respiratory distress syndrome, and polycythemia on mother and fetus [6]. On the other hand, the risk of developing type 2 diabetes, metabolic syndrome, and cardiovascular problems will increase in the mother with GDM and her child in the future [7]. GDM is also a serious concern for any system with increasing use of health and care resources and adverse outcomes, many of which can be mitigated by early diagnosis and treatment [8]. GDM is associated with physiological changes in the lipid profile of pregnant women [9]. A lipid profile is a direct measure of total cholesterol (TC), triglyceride (TG), high-density lipoprotein cholesterol (HDL-C), low-density lipoprotein cholesterol (LDL-C), and very low-density lipoprotein cholesterol (VLDL-C) [10]. During early pregnancy, the increase in maternal fat depots is facilitated by insulin, followed by increased adipose tissue breakdown, and subsequent hypertriglyceridemia, mainly due to insulin resistance and estrogen effects [11]. It is known that many factors affect lipid levels in GDM because carbohydrate metabolism directly affects lipid metabolism. There is still controversy over the association between lipid profile and GDM [12]. Although lipid levels have been extensively studied during pregnancy, there are conflicting results in this regard. There are also few studies on whether fat patterns are different in women with GDM in the first trimester of pregnancy [9]. Since changes in fat metabolism during pregnancy can be associated with adverse pregnancy outcomes such as GDM, this comprehensive systematic review and meta-analysis aimed to determine the effect of GDM on lipid profile and this study was performed to update the previous results and find reliable data in order to complete the existing knowledge.

## 2 Materials and methods

Preferred Reporting Items for Systematic Reviews and Meta-Analyses (PRISMA) guidelines were observed in the report of the study. PRISMA contains 27 items related to the content of a systematic and meta-analysis, and includes abstracts, methods, results, discussions, and financial resources [13,14,15]. This study was approved by ethnical code IR.ABZUMS.REC.1399.140.

## 3 Information source and search strategy

PubMed, Web of Science, Scopus, Google Scholar ProQuest, and ClinicalTrials were searched until 2021 by MESH keywords and search strategy was as below:

1. ‘Gestational diabetes’[tiab], OR ‘GD’ [tiab], OR ‘Gestational Diabetes Mellitus’ [tiab], OR ‘GDM’[tiab], OR ‘pregnancy induced diabetes’[tiab], ‘Diabetes, Pregnancy-Induced’[tiab], ‘Diabetes, Pregnancy Induced’[tiab], ‘Diabetes Mellitus, Gestational’[tiab]
2. ‘lipid profile’[tiab], OR ‘total cholesterol’[tiab], OR ‘high-density lipoprotein-cholesterol’[tiab], OR ‘low-density lipoprotein-cholesterol’[tiab], OR ‘Very low-density lipoprotein-cholesterol’[tiab], OR ‘triglycerides’[tiab], OR ‘TC’[tiab], OR ‘LDL-C’[tiab], OR ‘HDL-C’[tiab], OR ‘VLDL-C’ [tiab], OR ‘TG’[tiab]
3. ‘Screening’[tiab], OR ‘Predicting’[tiab]
4. 1 AND 2
5. 1 AND 3
6. 1 AND 2 AND 3

## 4 Eligibility criteria

### 4.1 Inclusion and exclusion criteria

Studies were included if they were published until 2021, full-text available, and with no language restrictions. Other inclusion criteria were: single pregnancy, GDM based on the criteria, and gestational age considered for each study based on ultrasound. Participation, intervention, comparators, outcomes, and study design (PICOS) criteria including:

Population: pregnant women

Exposure: serum lipid concentration

Comparison: healthy control group

Outcome: GDM

Study design: cohort, case control, and cross sectional

### 4.2 Exclusion criteria

Multiple pregnancies, smoking and alcohol use, a history of type 1 and type 2 diabetes, a history of pre-pregnancy hyperlipidemia, a history of hypertension/cardiovascular disease, a history of metabolic syndrome, a history of other systemic diseases such as liver failure, chronic renal failure, endocrine disorders, and autoimmune diseases. Case reports, qualitative, and review studies, as well as research with missing data, were also excluded.

### 4.3 Study selection

**Figure 1:**
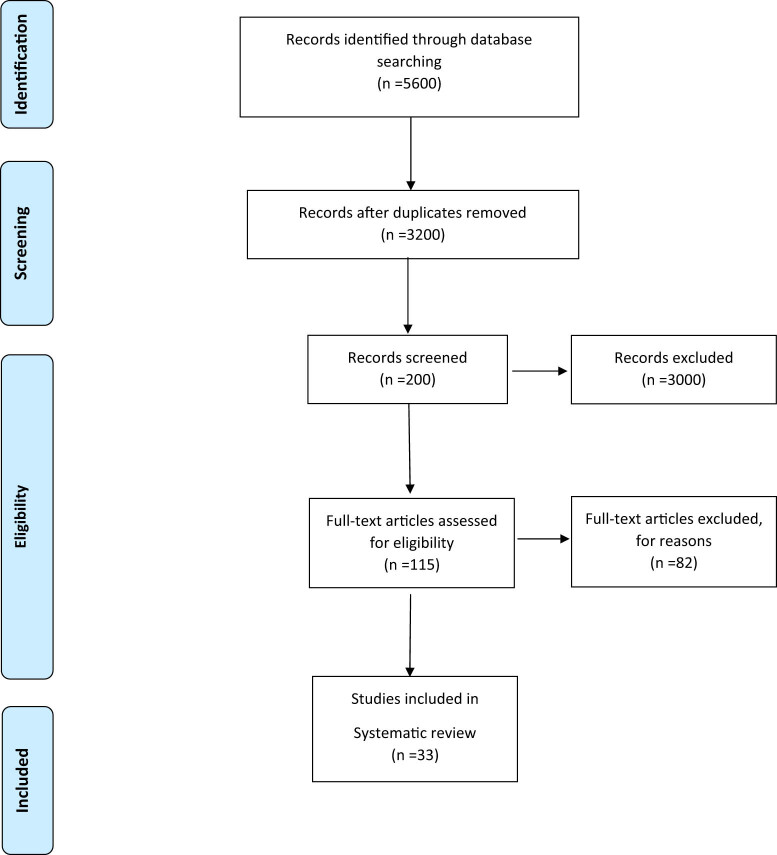
Flowchart of the study selection process.

The EndNote reference management software was applied to manage the acquired articles. The initial search yielded 5,600 results. The eligibility of these articles was independently evaluated by two authors and any disagreements were resolved by consensus. In the first stage 2,400 articles were excluded due to being irrelevant or duplicated. After reviewing the titles and abstracts of the remaining articles, 3,000 more papers were excluded. In the evaluation of the full texts, 82 out of the remaining 115 articles were excluded due to being ineligible. Finally, a total of 33 eligible articles were reviewed (Figure 1).

### 4.4 Quality assessment

Newcastle Ottawa scale was used to measure the quality of studies. This scale is used to measure the quality of cohort and case control studies. The validity and reliability of this tool have been proven in various studies [16,17].

## 5 Data extraction

Two authors independently performed the study selection and validity assessment and resolved any disagreements by consulting a third researcher. The first author name, year, study design, country, sample size, maternal age, maternal BMI or weight, diagnostic criteria, methods of analysis, quality assessment, gestational age at sampling, TC, LDL-C, HDL-C, TG, VLDL-C, TG/HDL-C ratio, and outcomes.

## 6 Unification of units

All lipid profiles were converted to mg/dL. For conversion of TC, HDL, and LDL from SI units mmol/L to mg/dL, the values were multiplied with 38.67. For conversion of TG from SI units mmol/L to mg/dL, the values were multiplied with 88.57. In order to calculation of VLDL, we used TG/5.

## 7 Statistical analysis

All analyses were conducted with Stata software version 14.0 (College Station, Texas). For each study, mean value and standard deviation (SD) of lipid profile were extracted and if IQR was reported we changed it to SD with IQR/1.35. Then, standardized mean difference (SMD) of lipids profile for each study was calculated based on Cohen’s d formula:

\begin{array}{l}\text{Cohen's}\hspace{.5em}\text{SMD}=\frac{{M}_{1}-\hspace{.25em}{M}_{2}}{{\text{SD}}_{\text{pooled}}},\\ \hspace{.5em}{\text{SD}}_{\text{pooled}}=\sqrt{\frac{({n}_{1}-1){\text{SD}}_{1}^{2}+\hspace{.25em}({n}_{2}-1){\text{SD}}_{2}^{2}}{{n}_{1}+{n}_{2}-2},}\end{array}]

where *M*_1,_ *n*_1,_ and SD_1,_ and *M*_2,_ *n*_2,_ and SD_2_ are mean values, samples size, and SDs in GDM and control groups, respectively. Some studies reported odds ratio (OR) and for calculating the SMD and standard error (Se), we used below formula:

\begin{array}{l}\text{SMD}=\frac{\sqrt{3}}{\text{π}}\hspace{.25em}\log \hspace{.25em}\text{OR,}\\ \text{Se}(\text{SMD})=\hspace{.25em}\frac{\sqrt{3}}{\text{π}}\text{Se}\hspace{.5em}(\text{log}\hspace{.5em}\text{OR}),\end{array}]

where log OR and π are the natural logarithm odds ratio and 3.14, respectively. Then, pooled SMD was calculated by “Metan” command [18]. Heterogeneity was determined using Cochran’s *Q* test of heterogeneity, and the *I* ^2^ index was used to quantify heterogeneity. In accordance with Higgins classification approach, *I* ^2^ values above 0.7 were considered as high heterogeneity. To estimate the pooled SMD for lipid profile and for subgroup analysis (based on trimester), the fixed-effect model was used, and when the heterogeneity was greater than 0.7, the random effects model was used. The meta-regression analysis was used to examine the effect of age, BMI, sample size, and publication date as factors affecting heterogeneity among studies. The “Meta bias” command [19] was used to check for publication bias, and if there was any publication bias, the pooled SMD was adjusted with the “Meta trim” command using the trim-and- fill method [20]. In all analyses, a significance level of 0.05 was considered [21].

## 8 Results

Finally, 33 studies with a sample size of 23,792 met the criteria for entering the meta-analysis (Table 1). Figure 1 also shows the flowchart of the study selection process. Serum lipid concentration between the groups with and without GDM of included studies is given in Table 2.

**Table 1:** The characteristics of included studies

Author (year)	SD	Country	SS		Maternal age (year)		BMI (kg/m^2^) or Weight (kg)		Diagnostic criteria of GDM	Method of analysis test	QAS
			GDM	Control	GDM	Control	GDM	Control			
Farsangi et al., 2020 [41]	CC	Iran	42	42	29.62 ± 0.80	27.54 ± 0.95	23.51 ± 3.37	22.80 ± 3.18	ADA	Enzymatic assay using commercial kits (Pars Azmun Inc, Iran).	8
Hossain et al., 2020 [42]	CS	Bangladesh	31	31	26.5	26.3			WHO	Enzymatic-colorimetric method	8
Saumya 2020 [43]	CS	India	51	149	32.29 *±* 4.42	30.15 *±* 4.33	—		IADPSG	HDL and TG were estimated by glucose oxidase–peroxidase method, cholesterol oxidase–peroxidase method (CHOD–POD), cholesterol oxidase–cholesterol esterase method, and glycerol phosphate oxidase method. Plasma LDL-C was calculated using Friedewald’s formula.	7
Wang et al., 2019 [9]	C	China	300	1,283	32.65 ± 3.92	31.53 ± 3.68	23.22 ± 3.49	21.87 ± 2.97	ADA	Automatic biochemical analyzer	8
Layton et al., 2019 [38]	C	Canada	67	739	30	31	27.0 (22.0–32.4)*	23.9 (21.5–27.5)*	IADPSG	Colorimetric method (Johnson & Johnson Clinical Diagnostics)	9
Correa et al., 2019 [26]	CC	Chile	16	80	32.63 ± 6.36	29.88 ± 5.75	26.55 (6.29)*	24.9 (4.2)*	IADPSG	VITROS Chemistry Products CHOL Slides, ©Ortho-Clinical Diagnostics, Inc., Buckinghamshire, UK	9
Aydemir et al., 2019 [40]	CC	Turkey	99	98	33.39 ± 4.92	32.29 ± 4.62	31.09 ± 4.841	27.91 ± 3.99	Department of Obstetrics and Gynecology, Cerrahpasa Medical Faculty, Istanbul University, Istanbul, Turkey	Automated latex-enhanced immunoassay	8
Anjum et al., 2019 [28]	CS	Saudi Arabia	25	50	31.4 ± 6.06	29.7 ± 6.12	NR	NR	ADA	Colorimetric method	7
Alyas et al., 2019 [44]	CC	Pakistan	58	100	18–40	NR	NR	ADA	Clinical chemistry analyzer	7	
Yue and Ying, 2019 [45]	CC	China	88	456	31.86 ± 0.47	29.98 ± 0.21	23.97 ± 0.5	21.22 ± 0.14	ACOG	Automatic biochemical analyzer	9
Zebunnesa et al., 2018 [46]	CS	Bangladesh	30	30	28.70 ± 3.95	28.76 ± 5.47	NR	NR	IADPSG	Multisystem automatic analyzer	8
Cao et al., 2018 [47]	CC	China	33	33	29.20 ± 1.03	28.70 ± 1.16	34	29	ADA	Automatic biochemistry analyzer	8
Bukowiecka-Matusiak et al., 2018 [39]	CC	Poland	32	11	31.0 (28–35)*	29.0 (28–30)	23.7 (21.4–26.3)*	20.9 (20.4–21.3)*	WHO	NR	8
Bugatto et al., 2018 [48]	C	Spain	22	23	31.4 + 6.0	30.5 + 4.5	26.6 + 6.0	25.2 + 6.5	National Diabetes Data Group	Modular DPD biochemical auto-analyzer	9
Barat et al., 2018 [49]	CS	Iran	250	87	30.49 ± 4.00	27.33 ± 4.87	28.5 ± 3.73	25.72 ± 4.33	ADA	Ziestchem Diagnostic Tehran	8
Bao et al., 2018 [50]	CC	USA	107	214	18–40	19–45			ACOG	Enzymatic assays using Roche COBAS 6000 Chemistry Analyzer	8
Pazhohan, et al., 2017 [51]	C	Iran	176	778	27.47 ± 3.54	24.23 ± 3.21	26.84 ± 3.87	24.28 ± 3.04	IADPSG	NR	8
Wang et al., 2017 [52]	C	China	5,218	—	28.52 ± 3.86	—	21.59 ± 3.23	—	Chinese criteria	NR	8
Ghodke et al., 2017 [53]	C	India	200	—	24.87 ± 2.7	—	NR	NR	NR	AU480 biochemistry auto analyzer by CHOD–POD method	7
Chen et al., 2017 [24]	CC	China	28	56	33.0 (30.3, 36.0)*	30.0 (28.0, 33.0)*	20.6 ± 2.5	20.1 ± 2.2	IADPSG	Particle number analysis method	8
Wang et al., 2016 [54]	CC	China	1,062	4,203	29.46 ± 3.96	28.29 ± 3.79	22.52 ± 3.36	21.33 ± 3.03	IADPSG	NR	8
Shen et al., 2016 [55]	C	China	188	1,122	30.56 ± 3.47	29.55 ± 3.13	22.07 ± 2.93	20.79 ± 2.9	IADPSG	Automatic biochemical analyzer	9
Liang et al., 2016 [56]	CC	China	55	50	28.2 ± 5.1	27.1 ± 5.4	22.7 ± 1.7	22.1 ± 2.1	National Diabetes Data Group	Tinder enzymatic method	8
Khosrowbeygi et al., 2016 [57]	CS	Iran	30	30	32.63 ± 0.72	28.53 ± 0.94	25.00 ± 0.23	24.84 ± 0.28	ADA	Available photometric methods	8
Jin et al., 2016 [58]	CS	China	934	—	29.21 ± 3.76	—	20.66 ± 2.70	—	IADPSG	Automatic biochemical analyzer	8
Han et al., 2016 [59]	CC	USA	254	490	27.8 ± 5.5	27.9 ± 5.2	26.1 ± 6.5	23.7 ± 4.6	Carpenter and Coustan	Kodak Ektachem Chemistry analyzer	7
Ertug et al., 2016 [60]	CS	Turkey	29	20	32 ± 4	27 ± 5	27.6 (25.5–29.9)	26.0 (23.5 – 28.0)	Carpenter and Coustan	Standard enzymatic colorimetric methods	8
Wang et al., 2015 [61]	CS	China	110	526	31 (29–34)*	29 (27–31)*	21.02 (19.24–22.56)*	20.03 (18.59–21.55)*	Ministry of Health China	Automatic chemistry analyzer	8
Li et al., 2015 [62]	C	China	379	2,166	31.60 ± 4.25	30.40 ± 7.36	22.57 + 4.75	20.81 + 5.45	ADA	End-point colorimetric method	7
dos Santos-Weiss et al., 2013 [63]	CC	Brazil	288	288	33.1 (30.0–37.0)*	32.5 (28.0–34.0)*	33.4 ± 6.4	26.1 ± 4.7	ADA	Automated system Architect Ci8200	9
Khan et al., 2012 [64]	CS	Pakistan	103	97	≥30	≥30	≥25		IADPSG	Enzymatic methods, enzymatic analysis in supernatant fraction, Friedewald’s equation	7
Caglar et al., 2012 [65]	CC	Turkey	19	15	30.3 ± 5.4	30.0 ± 4.7	65.7 ± 9.1	64.5 ± 9.3	ADA	Enzymatic colorimetric assays	8
Wiznitzer et al., 2009 [66]	CS	Israel	1,209	8,700	30.9 ± 6.5	29.5 ± 5.8	NR	NR	Universal screening	NR	7
McGrowder et al., 2009 [67]	CC	India	84	94	30.18 ± 0.88	29.61 ± 1.03	NR	NR	WHO	Multichannel auto analyzer	7

*Median (IQR), Abbreviations: SD: study design, SS: sample size, QAS: quality assessment, CC: case control, CS: cross sectional, C: cohort, BMI: body mass index, GDM: gestational diabetes mellitus, ADA: American Diabetes Association, IADPSG: International Association of Diabetes and Pregnancy Study Groups, WHO, World Health Organization, ACOG: American College of Obstetricians and Gynecologists, NR: not reported.

**Table 2:** Serum lipid concentration between the groups with and without GDM of included studies

Author, year	GA at sampling (week)	TC		LDL-C		HDL-C		TG		VLDL		TG/HDL-C ratio		Out come
		GDM	Control	GDM	Control	GDM	Control	GDM	Control	GDM	Control	GDM	Control	
Farsangi et al., 2020 [41]	T3	228.96 ± 52.03 mg/dL	211.59 ± 41.83 mg/dL	122.41 ± 4.82 mg/dL	144.54 ± 26.01 mg/dL	53.10 ± 1.72 mg/dL	46.64 ± 1.70 mg/dL	225.58 ± 89.849 mg/dL	208.38 ± 80.66 mg/dL	NR	NR	NR	NR	Significant for HDL
Hossain et al., 2020 [42]	T2–T3	194.21 ± 42.18 mg/dL	208.52 ± 42.18 mg/dL	109.25 ± 28.80 mg/dL	119.30 ± 34.76 mg/dL	47.50 ± 16.17 mg/dL	47.18 ± 11.71 mg/dL	204.78 ± 58.50 mg/dL	202.34 ± 79.18 mg/dL	NR	NR	NR	NR	NS
Saumya, 2020 [43]	T1	0.07 [ 0.04, 0.11]*		0.12[0.09,0.16]*		NR		0.21[0.18,0.24]*		NR		NR		Significant
Wang et al., 2019	T1,T2, and T3	T1: 157.36 ± 25.90, T2: 218.45 ± 38.66, T3: 233.53 ± 41.37 mg/dL	T1: 156.21 ± 23.41, T2: 223.87 ± 35.41, T3: 238.95 ± 41.37 mg/dL	T1: 85.46 ± 21.27, T2: 114.46 ± 31.71, T3: 237.05 ± 34.42 mg/dL	T1: 82.75 ± 21.65,T2: 117.94 ± 29.78,T3: 128.77 ± 35.58 mg/dL	T1: 52.98 ± 10.05,T2: 67.67 ± 12.76,T3: 63.42 ± 11.60 mg/dL	T1: 55.30 ± 10.44,T2: 72.31 ± 13.35,T3: 66.90 ± 12.76 mg/dL	T1: 95.62 ± 50.46,T2: 227.55 ± 100.05,T3: 297.50 ± 133.70 mg/dL	T1: 80.57 ± 44.27, T2: 197.44 ± 82.34, T3: 272.70 ± 108.90 mg/dL	T1: 19.12 ± 10.09, T2: 45.51 ± 20.01, T3: 59.50 ± 26.74 mg/dL	T1: 16.11 ± 8.85, T2: 39.49 ± 16.47, T3: 26.74 ± 54.54 mg/dL	T1: 0.84 ± 0.54,T2: 1.58 ± 0.96,T3: 2.20 ± 1.45	T1: 0.66 ± 0.44, T2: 1.27 ± 0.80, T3: 1.90 ± 1.08	Significant for TG, HDL, and TG/HDL-C ratio
Layton et al., 2019	T2	235.85 ± 26.34 mg/dL	238.95 ± 40.38 mg/dL	126.06 ± 17.18 mg/dL	131.86 ± 34.37 mg/dL	85.07 ± 14.60 mg/dL	73.47 ± 15.75 mg/dL	154.95 ± 16.40 mg/dL	161.14 ± 49.62 mg/dL	30.99 ± 3.28 mg/dL	32.23 ± 9.92 mg/dL	NR	NR	Significant for TG
Correa et al., 2019	T1	193.01 ± 38.74 mg/dL	165.50 19.11 mg/dL	116.40 ± 35.08 mg/dL	91.80 ± 20.37 mg/dL	60 ± 8.52 mg/dL	66 ± 12.96 mg/dL	137.50 ± 48.15 mg/dL	96.50 ± 32.22 mg/dL	27.50 ± 9.63 mg/dL	19.30 ± 6.44 mg/dL	NR	NR	Significant for TC, TG, and LDL
Aydemir et al., 2019	T3	242.86 ± 37.57 mg/dL	229.14 ± 44.22 mg/dL	140.82 ± 37.04 mg/dL	143.86 ± 30.07 mg/dL	62.48 ± 13.54 mg/dL	62.34 ± 12.30 mg/dL	203.30 ± 75.09 mg/dL	197.13 ± 74.6 mg/dL	40.66 ± 15.02 mg/dL	39.43 ± 14.92 mg/dL	NR	NR	Significant for TC
Anjum et al., 2019	T2	185.12 ± 22.78 mg/dL	197.40 ± 40.53 mg/dL	111.73 ± 17.26 mg/dL	114.22 ± 35.64 mg/dL	49.00 ± 8.54 mg/dL	62.12 ± 15.32 mg/dL	122.52 ± 51.50 mg/dL	105.26 ± 41.70 mg/dL	24.50 ± 10.30 mg/dL	21.05 ± 8.34 mg/dL	2.70 ± 1.6	1.76 ± 0.90	Significant for HDL-C and TG/HDL
Alyas et al., 2019	T1 and T2	T1: 308.91 ± 1.27,T2: 367.86 ± 2.39 mg/dL	T1: 287.71 ± 1.67,T2: 340.43 ± 1.58 mg/dL	T1: 165.62 ± 2.02,T2: 227.13 ± 3.43 mg/dL	T1: 131.16 ± 1.02,T2: 201.60 ± 2.75 mg/dL	T1: 45.71 ± 0.74,T2: 33.42 ± 1.93 mg/dL	T1: 59.80 ± 0.78,T2: 41.63 ± 0.87 mg/dL	T1: 369.52 ± 3.34,T2: 450.45 ± 4.21 mg/dL	T1: 346.42 ± 3.52,T2: 423.94 ± 3.38 mg/dL	T1: 39.95 ± 0.95,T2: 54.39 ± 1.11 mg/dL	T1: 31.88 ± 0.30,T2: 47.38 ± 0.48 mg/dL	NR	NR	Significant
Yue and Ying, 2019	T2	239.72 ± 44.80 mg/dL	238.56 ± 23.20 mg/dL	141.53 ± 1.55 mg/dL	143.08 ± 1.55 mg/dL	53.36 ± 0.77 mg/dL	53.36 ± 0.39 mg/dL	397.54 ± 14.16mg/dL	332.02 ± 7.97 mg/dL	79.51 ± 2.83 mg/dL	66.40 ± 1.59 mg/dL	3.25 ± 0.12	2.77 ± 0.07	Significant for TG and TG/HDL
Zebunnesa et al., 2018	T3	209.53 ± 34.66 mg/dL	230.45 ± 45.25 mg/dL	119.86 ± 31.56 mg/dL	110.22 ± 24.79 mg/dL	55.63 ± 34.26 mg/dL	53.02 ± 6.81 mg/dL	267.96 ± 56.34 mg/dL	232.88 ± 58.43 mg/dL	53.59 ± 11.27 mg/dL	46.58 ± 11.69 mg/dL	NR	NR	Significant for TG and TC
Cao et al., 2018	T3	146.92 ± 19.15 mg/dL	80.42 ± 41.64 mg/dL	146.95 ± 16.92 mg/dL	108.28 ± 15.14 mg/dL	65.74 ± 14.63 mg/dL	81.20 ± 13.64 mg/dL	557.80 ± 16.41 mg/dL	283.33 ± 19.21 mg/dL	44.07 ± 5.41 mg/dL	46.4 ± 6.14 mg/dL	NR	NR	Significant
Bukowiecka-Matusiak et al., 2018	T2	259.9 ± 37.11 mg/dL	219.5 ± 37.40 mg/dL	141.0 ± 42.22 mg/dL	119.0 ± 25.18 mg/dL	74.10 ± 21.40 mg/dL	61.4 ± 9.77 mg/dL	215.9 ± 63.70 (mg/dL)	157.6 ± 64.74 mg/dL	43.18 ± 12.74 mg/dL	31.52 ± 12.95 mg/dL	NR	NR	Significant for TG and TC
Bugatto et al., 2018	T3	249.4 + 44.8 mg/dL	256.9 + 42.8 mg/dL	143.1 + 38.0 mg/dL	146.1 + 35.8 mg/dL	65.4 + 18.6 mg/dL	70.8 + 21.9 mg/dL	252.0 + 82.7 mg/dL	191.4 + 68.8 mg/dL	50.40 ± 16.54 mg/dL	38.28 ± 13.76 mg/dL	NR	NR	Significant for TG
Barat et al., 2018	T3	228.82 ± 41.10 mg/dL	234.41 ± 132.01 mg/dL	122.82 ± 31.47 mg/dL	122.57 ± 43.35 mg/dL	53.30 ± 14.88 mg/dL	66.28 ± 25.78 mg/dL	275.43 ± 69.33 mg/dL	205.53 ± 72.51 mg/dL	55.09 ± 13.87 mg/dL	41.11 ± 14.50 (mg/dL)	5.37 ± 1.56	3.38 ± 1.54	Significant for TG, HDL, and TG/HDL
Bao et al., 2018	T1 and T2	T1: 185.01 ± 16.14, T2: 195.10 ± 22.41 mg/dL	T1: 179 ± 19.54, T2: 208 ± 18.41 mg/dL	T1: 90 ± 0.41, T2: 98 ± 10.24 mg/dL	T1: 88 ± 10.41, T2: 105 ± 11.67 mg/dL	T1: 57.3 ± 9.87, T2: 63.3 ± 13.89 mg/dL	T1: 62.3 ± 21.71, T2: 72.3 ± 13.04 mg/dL	T1: 155 ± 11.41, T2: 198 ± 13.20 mg/dL	T1: 119 ± 19.10, T2: 207 ± 25.19 mg/dL	T1: 31 ± 2.28, T2: 39.60 ± 2.64 mg/dL	T1: 23.80 ± 3.82, T2: 41.40 ± 5.04 mg/dL	NR	NR	Significant for TG and HDL
Pazhohan, 2017	T1	202.9 ± 31.83 mg/dL	195.9 ± 30.0 mg/dL	NR	NR	NR	NR	198.3 ± 105.6 mg/dL	164.1 ± 44.3 mg/dL	39.66 ± 21.12 mg/dL	32.82 ± 8.86 mg/dL	3.84 ± 0.83	3.14 ± 0.44	Significant for TG and TG/HDL
Wang et al., 2017	T1	177.50 ± 33.26 mg/dL	171.70 ± 30.16 mg/dL	92.80 ± 27.84 mg/dL	88.55 ± 25.13 mg/dL	65.35 ± 20.50 mg/dL	67.29 ± 16.63 mg/dL	117.80 ± 63.77 mg/dL	103.63 ± 59.34 mg/dL	23.56 ± 12.75 mg/dL	20.73 ± 11.87 mg/dL	NR	NR	Significant
Ghodke et al., 2017	T2 and T3	T2: 223.50 ± 25.16, T3: 242.83 ± 27.14 mg/dL	T2: 214.60 ± 14.11, T3: 242.65 ± 14.19 mg/dL	T2: 96.83 ± 31.39, T3: 150.16 ± 9.88 mg/dL	T2: 92.41 ± 14.41, T3: 137.82 ± 10.41 mg/dL	T2: 52.00 ± 7.07, T3: 41.16 ± 7.27 mg/dL	T2: 49 ± 6.14, T3: 43.07 ± 5.74 mg/dL	T2: 214.33 ± 18.64, T3: 230.50 ± 17.03 mg/dL	T2: 186.68 ± 12.41, T3: 216.78 ± 16.44 mg/dL	T2: 34 ± 5.65, T3: 30.58 ± 5.83 mg/dL	T2: 36.27 ± 3.98, T2: 32.25 ± 4.02 mg/dL	NR	NR	Significant for TG
Chen et al., 2017	T2	222.96 ± 36.21 mg/dL	240.59 ± 42.69 mg/dL	96.61 ± 28.65 mg/dL	115.00 ± 35.78 mg/dL	79.43 ± 17.35 mg/dL	84.79 ± 18.96 mg/dL	219.5 (175.8, 285.3) mg/dL	185.0 (146.5, 236.0) mg/dL	43.90 ± 16.22 mg/dL	37 ± 13.26 mg/dL	2.96 (2.14, 3.84)	2.16 (1.64, 3.10)	Significant for LDL and TG/HDL
Wang et al., 2016	T1	176.69 ± 32.09 mg/dL	171.67 ± 30.16 mg/dL	NR	NR	NR	NR	191.32 ± 1.22 mg/dL	103.60 ± 80.57 mg/dL	30.26 ± 0.24 mg/dL	20.72 ± 16.11 mg/dL	0.92 ± 1.61	0.71 ± 0.46	Significant
Shen et al., 2016	T1,T2, and T3	T1: 196.41 ± 15.41, T2: 239.72 ± 16.47, T3: 259.83 ± 16.97 mg/dL	T1: 190.61 ± 12.64, T2: 239.72 ± 10.75, T3: 268.72 ± 13.64 mg/dL	T1: 134.18 ± 19.41, T2: 157 ± 13.64, T3: 169.37 ± 20.97 mg/dL	T1: 129.16 ± 17.85, T2: 159.70 ± 16.95, T3: 177.50 ± 17.68 mg/dL	T1: 65.35 ± 19.65, T2: 72.70 ± 13.17, T3: 71.54 ± 18.32 mg/dL	T1: 65.74 ± 15.65, T2: 73.47 ± 16.98, T3: 73.09 ± 19.54 mg/dL	T1: 136.35 ± 16.39, T2: 233.74 ± 18.31, T3: 285.98 ± 21.39 mg/dL	T1: 115.98 ± 16.74, T2: 201.87 ± 9.47, T3: 264.73 ± 9.87, mg/dL	T1: 27.27 ± 3.28, T2: 46.75 ± 3.66, T3: 57.20 ± 4.28 mg/dL	T1: 23.20 ± 3.35, T2: 40.37 ± 1.89, T3: 52.95 ± 1.97 mg/dL	NR	NR	Higher TG and LDL-C at T1, but lower at T2 and T3
Liang et al., 2016	T2	266.78 ± 81.19 mg/dL	177.86 ± 65.73 mg/dL	NR	NR	NR	NR	513.33 ± 123.95 mg/dL	239.06 ± 61.98 mg/dL	102.71 ± 24.79 mg/dL	47.81 ± 12.40 mg/dL	NR	NR	Significant
Khosrowbeygi et al., 2016	T2	234.90 ± 11.51 mg/dL	256.13 ± 12.56 mg/dL	142.25 ± 12.66 mg/dL	149.27 ± 9.70 mg/dL	36.90 ± 3.25 mg/dL	62.07 ± 2.18 mg/dL	278.73 ± 23.17 mg/dL	223.97 ± 18.51 mg/dL	55.75 ± 4.63 mg/dL	44.79 ± 3.70 mg/dL	8.64 ± 0.76	3.65 ± 0.31	Significant for HDL-C and TG/HDL-C
Jin et al., 2016	T1,T2, and T3	T1: 152.72 ± 26.92, T2: 179.79 ± 25.20, T3: 242.42 ± 43.24 mg/dL	—	T1: 87 ± 10.60, T2: 95.13 ± 1.67, T3: 110.98 ± 32.36 mg/dL	—	T1: 64.19 ± 22.50, T2: 64.58 ± 9.63, T3: 69.60 ± 13.33 mg/dL	—	T1: 194.79 ± 62.96, T2: 216.92 ± 51.15, T3: 270.93 ± 105.59 mg/dL	—	T1: 38.96 ± 12.59, T2: 43.38 ± 10.23, T3: 54.19 ± 21.12 mg/dL	NR	NR	NR	Significant for TG, LDL-C, and HDL-C
Han et al., 2016	T2–T3	182.9 ± 33.3 mg/dL	176 ± 32.6 mg/dL	371.7 ± 125.5 mg/dL	386.8 ± 119.9 mg/dL	4180.4 ± 1524.9 mg/dL	4650.8 ± 1605.5 mg/dL	NR	NR	134.4 ± 44.5 mg/dL	130.3 ± 43.5 (mg/dL)	NR	NR	Significant for HDL and TC
Ertug et al., 2016	T2	234 ± 46 mg/dL	241 ± 54 mg/dL	124 ± 41 mg/dL	141 ± 52 mg/dL	64 ± 13 mg/dL	69 ± 16 mg/dL	220 ± 78 mg/dL	160 ± 49 mg/dL	44 ± 15.60 mg/dL	32 ± 9.80 mg/dL	NR	NR	Significant for TG and HDL
Wang et al., 2015	T3	NR	NR	NR	NR	69.99 ± 16.90 mg/dL	72.70 ± 13.46 mg/dL	193.01 ± 64.27 mg/dL	172.65 ± 54.44 mg/dL	38.60 ± 12.85 mg/dL	34.53 ± 10.89 mg/dL	1.24 ± 0.63	1.04 ± 0.43	Significant for TG and TG/HDL
Li et al., 2015	T1	185.20 ± 41.75 mg/dL	176.31 ± 31.70 mg/dL	84.30 ± 27.84 mg/dL	80.82 ± 22.81 mg/dL	71.15 ± 17.79 mg/dL	76.18 ± 19.33 mg/dL	142.54 ± 77.91 mg/dL	111.56 ± 55.78 mg/dL	28.51 ± 15.58 mg/dL	22.31 ± 11.16	NR	NR	Significant
dos Santos-Weiss et al., 2013	T1,T2, and T3	T1: 193.32 ± 38.66, T2: 216.52 ± 46.39, T3: 233.92 ± 39.51 mg/dL	T1: 185.59 ± 34.80, T2: 228.12 ± 46.40, T3: 241.65 ± 50.26 mg/dL	T1: 96.67 ± 34.37, T2: 100.54 ± 37.22, T3: 129.54 ± 35.80 mg/dL	T1: 108.28 ± 25.77, T2: 143.08 ± 40.10, T3: 137.27 ± 32.94 mg/dL	T1: 46.40 ± 11.60, T2: 58.01 ± 11.60, T3: 56.07 ± 11.60 mg/dL	T1: 54.14 ± 15.47, T2: 61.87 ± 15.47, T3: 61.87 ± 17.40 mg/dL	T1: 221.35 ± 146.60, T2: 194.79 ± 59.26, T3: 230.20 ± 72.22 mg/dL	T1: 97.39 ± 32.59, T2: 150.51 ± 51.85, T3: 172.65 ± 58.82 mg/dL	T1: 44.27 ± 29.32, T2: 38.96 ± 11.85, T3: 46.01 ± 14.44 mg/dL	T1: 19.48 ± 6.52, T2: 30.10 ± 10.37, T3: 34.53 ± 11.76 mg/dL	NR	NR	Significant
Khan et al., 2012	T3	206 ± 18.79 mg/dL	195 ± 24.15 mg/dL	93 ± 18.71 mg/dL	88 ± 16.35 mg/dL	55 ± 8.20 mg/dL	56 ± 8.82 mg/dL	190 ± 19.83 mg/dL	172 ± 21.66 mg/dL	38 ± 3.97 mg/dL	34.40 ± 4.33 mg/dL	NR	NR	Significant for TC and TG
Caglar et al., 2012	T2	239.8 ± 39.7 mg/dL	232.2 ± 36.7 mg/dL	138.9 ± 42.1	135.6 ± 31.0 mg/dL	67.5 ± 13.7 mg/dL	75.3 ± 20.3 mg/dL	207.9 ± 66.8 mg/dL	191.1 ± 60.7 mg/dL	41.58 ± 13.36 mg/dL	38.22 ± 12.14 mg/dL	NR	NR	Not significant
McGrowder et al., 2009	T3	220.77 ± 9.28 mg/dL	193.71 ± 12.37 mg/dL	128.38 ± 9.28 mg/dL	117.94 ± 13.15 mg/dL	48.34 ± 3.10 mg/dL	56.07 ± 3.10 mg/dL	162.02 ± 8.85 mg/dL	126.61 ± 17.71 mg/dL	13.14 ± 0.77 mg/dL	12.75 ± 1.93 mg/dL	1.24 ± 0.08	1.21 ± 0.20	Significant for TC and TG

*Odds ratio (OR). Abbreviations: GA, gestational age; GDM, gestational diabetes mellitus; HDL-C, high-density lipid cholesterol; LDL-C, low-density lipid cholesterol; TC, total cholesterol; TG, triglycerides; NS, not significant, NR: not reported.

## 9 Pooled SMD

**Figure 2:**
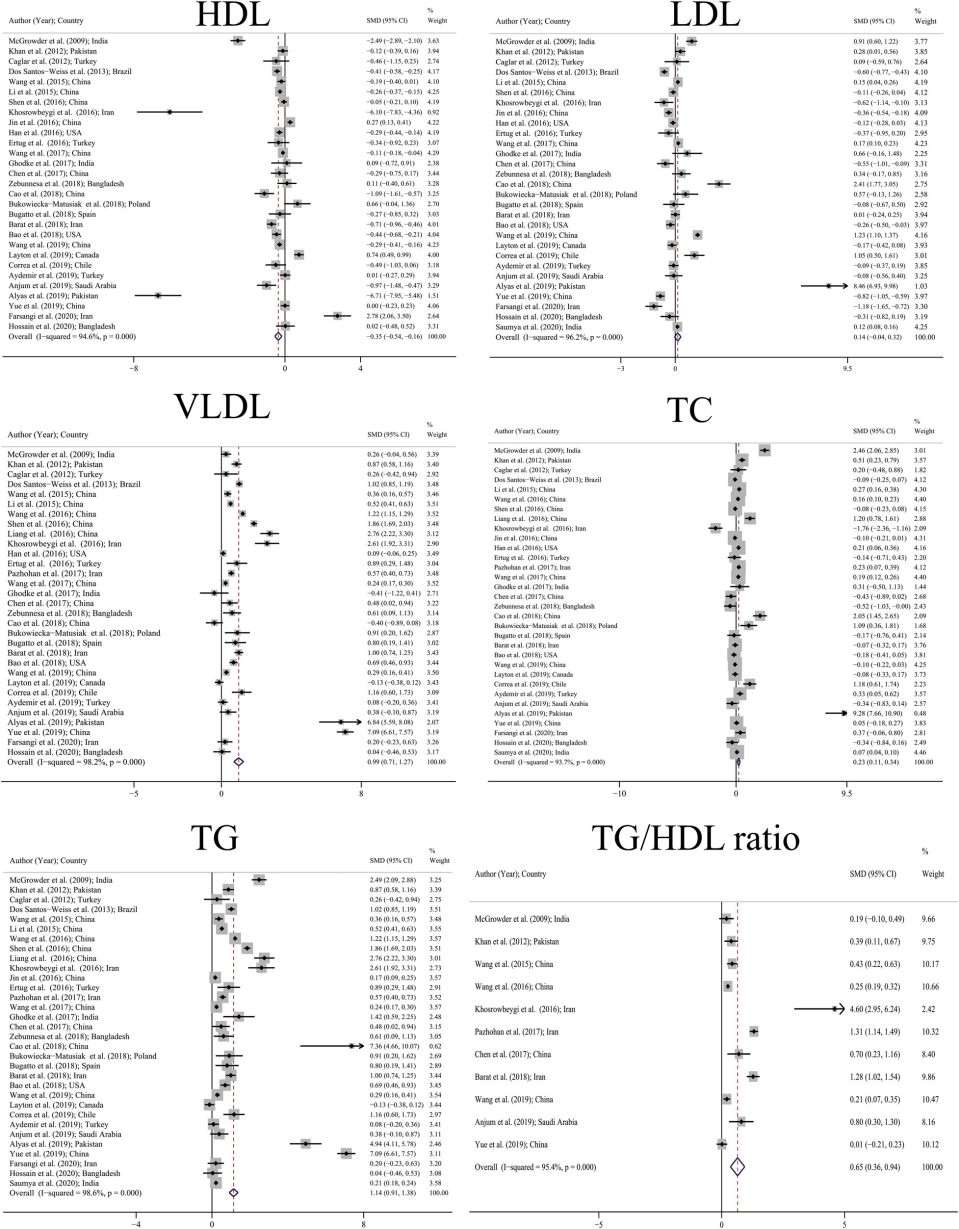
Pooled SMD of lipid profile based on random effects model. The midpoint of each line segment shows the SMD, the length of the line segment indicates 95% confidence interval in each study, and the diamond mark illustrates the pooled SMD for different lipid profile.

Table 3 shows the pooled SMD and Figure 2 shows the forest plot for the pooled SMD including TC, LDL, HDL, TG, VLDL, and TG/HDL ratio. Accordingly, there were 32 studies for TC, 29 studies for LDL, 29 studies for HDL, 32 studies for TG, 31 studies for VLDL, and 11 studies for TG/HDL ratio. As is clear from the forest plot, pooled SMD for TC and TG was 0.23 mg/dL (95% CI: 0.11–0.34) and 1.14 mg/dL (95% CI: 0.91–1.38). In other words, the mean values of TC and TG in people with GDM were higher than that in normal people. A similar pattern was observed for the VLDL and TG/HDL ratio, with pooled SMD for the VLDL and TG/HDL ratios 0.99 mg/dL (95% CI: 0.71–1.27) and 0.65 mg/dL (95% CI: 0.36–0.94), respectively, which indicates that the average of these indices was higher in the GDM group. Pooled SMD for HDL was also −0.35 mg/dL (95% CI: −0.54 to −0.16). In other words, in general, people with GDM had a mean HDL lower than normal people. Although pooled SMD was higher for LDL in the GDM group, this difference was not significant (0.14 [95% CI: −0.04 to 0.32]). Of all lipid profiles, the biggest difference between the GDM and control groups was observed in TG (SMD: 1.14).

**Table 3:** Result of meta-analysis for calculation of lipid profile SMD; publication bias and fill and trim method

Lipids profile	Meta-analysis			Egger’s test for publication bias		Fill and trim	
	Number	I^2^%	SMD	Coefficient (95% CI)	*P*-value	SMD	95% CI
TC	32	93.7	0.23 (0.11–0.34)	1.24 (−0.60–3.10)	0.179	—	—
LDL	29	96.2	0.14 (−0.04 to 0.32)*	−0.05 (−2.75–2.66)	0.972	—	—
HDL	29	94.6	−0.35 (−0.54 to −0.16)	−1.77 (−4.37–0.08)	0.173	—	—
TG	32	98.6	1.14 (0.91–1.38)	5.21 (1.70–8.71)	0.005	1.13	(0.92–1.39)
VLDL	31	98.2	0.99 (0.71–1.27)	2.04 (−2.23–6.31)	0.337	—	—
TG/HDL ratio	11	95.4	0.65 (0.36–0.94)	3.58 (−1.29–8.46)	0.130	—	—

*No significance; SMD: standardized mean difference; TC: total cholesterol; TG: triglyceride; CI: confidence interval; LDL: low-density lipoproteins; HDL: high-density lipoproteins; VLDL: very low-density lipoproteins.

## 10 Pooled SMD based on different trimesters

**Figure 3:**
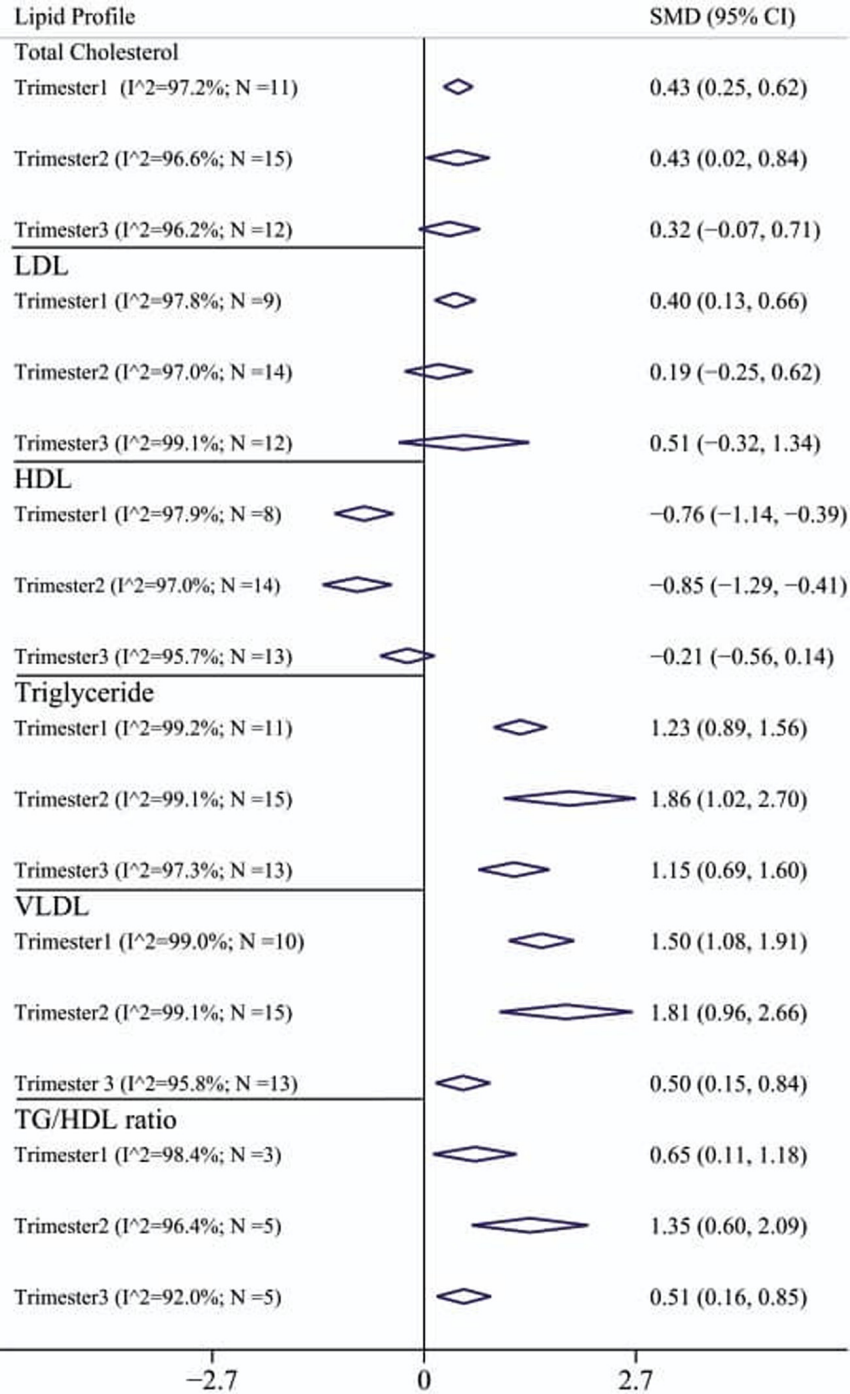
Pooled SMD and 95% confidence interval of lipid profile based on different trimesters.

Figure 3 shows the pooled SMD values for the lipid profile in terms of trimester. Accordingly, pooled SMD for TG, VLDL, and TG/HDL ratio at different trimesters in GDM group was significantly higher than that in normal individuals. In contrast, pooled SMD for HDL in 1st trimester (−0.76 [95% CI: −1.14 to −0.39]) and 2nd trimester (0.85 [95% CI: −1.29 to −0.41]) in the GDM group were significantly lower than that in normal group, and in the 3rd trimester no difference was observed between the two groups. Pooled SMD for LDL was significantly different only in the 1st trimester (0.40 [95% CI: 0.13–0.66]) so that in the GDM group the mean LDL was higher than that in the control group, and for the 2nd trimester (0.19 [95% CI: −0.25 to 0.62]) and for the third trimester (0.51 [95% CI: −0.32 to 1.34]), no significant difference was observed. Also, pooled SMD for TC only in the 1st trimester (0.43 [95% CI: 0.25–0.62]) and the second trimester (0.43 [95% CI: 0.02–0.84]), there was a significant difference between the two groups and in the 3rd trimester, a significant difference was not observed.

## 11 Publication bias

Table 3 shows the publication bias results based on the Egger’s test and the fill and trim method. As it turns out, there was a significant publication bias for TG (coefficient; 5.21; *P*: 0.005). According to the fill and trim method, the value of adjusted pooled SMD for TG was 1.13 (95% CI: 0.92–1.39), which was not significantly different from the pooled SMD calculated for TG (1.14 [95% CI: 0.91–1.38]). No publication bias was observed for other lipid profiles including TC, LDL, HDL, VLDL, and TG/HDL ratio.

## 12 Heterogeneity and meta-regression results

As shown in Table 3, there was significant heterogeneity between different studies for lipid profiles (Cochran’s *Q* test *P*-value < 0.001 for all lipid profiles) so that the *I* ^2^ index was above 90% for all lipid profiles. Table 4 shows the meta-regression results to investigate the effect of publication year, sample size, age, and BMI on heterogeneity between studies. Accordingly, none of the variables had a significant role on heterogeneity between studies (*P* > 0.05 for all of them).

**Table 4:** Results of the univariate meta-regression analysis on the heterogeneity of the determinants

Lipids profile	Publication year		Sample size		Mean age		BMI mean	
	Coefficient (95% CI)	*P*-value	Coefficient (95% CI)	*P*-value	Coefficient (95% CI)	*P*-value	Coefficient (95% CI)	*P*-value
TC	−0.015 (−0.25 to 0.22)	0.898	0.00 (−0.01 to 0.01)	0.642	−0.04 (−0.26 to 0.18)	0.701	0.00 (−0.04 to 0.04)	0.999
LDL	0.05 (−0.18 to 0.27)	0.688	0.01 (−0.01 to 0.01)	0.815	−0.06 (−0.29 to 0.17)	0.609	0.01 (−0.04 to 0.05)	0.750
HDL	0.11 (−0.14 to 0.35)	0.392	0.00 (−0.01 to 0.01)	0.551	0.00 (−0.25 to 0.25)	0.995	−0.02 (−0.09 to 0.05)	0.547
Triglyceride	0.00 (−0.25 to 0.25)	0.991	0.01 (−0.01 to 0.01)	0.551	−0.03 (−0.28 to 0.22)	0.794	−0.02 (−0.11 to 0.07)	0.631
VLDL	0.09 (−0.16 to 0.34)	0.464	0.00 (0.00 to 0.00)	0.736	0.01 (−0.23 to 0.24)	0.935	−0.03 (−0.11 to 0.04)	0.352
TG/HDL ratio	0.04 (−0.20 to 0.27)	0.742	0.01 (−0.01 to 0.01)	0.492	−0.06 (−0.26 to 0.15)	0.558	0.26 (−0.05 to 0.56)	0.086

CI: confidence interval; TC: total cholesterol; LDL: low-density lipoproteins; TG: triglyceride; HDL: high-density lipoproteins; VLDL: very low-density lipoproteins; BMI: body mass index.

## 13 Discussion

The aim of this comprehensive systematic review and meta-analysis was to determine the effect of GDM on lipid profile. In this study we have concluded the following: (1) the levels of TC, LDL-C, VLDL-C, and TG were higher in women with GDM than in normal pregnant women, (2) the level of HDL-C was lower in women with GDM than in normal pregnant women, and (3) of all lipid profiles, the largest difference between the GDM and control groups was observed in TG.

Studies have shown that even mild hyperglycemia during pregnancy is associated with an increase in perinatal complications [22,23]. Although the adverse effects of GDM on the mother and fetus are widely known, there are still many unresolved issues regarding GDM [24]. Therefore, the WHO states that there are many ambiguities about the various strategies for screening for GDM. However, despite recent research, there is still no general international agreement on the best way to screen for GDM, and screening for diabetes during pregnancy is essential because with timely diagnosis, appropriate treatment can be provided, and thereby, maternal and fetal complications, especially pre-eclampsia, macrosomia, and shoulder dystocia can be reduced [25]. In this regard, many researchers are interested in studying different markers in pregnant women so that they can detect the adverse effects of pregnancy, including diabetes, with the changes in these markers and reduce the complications [26]. Various markers including C-reactive protein, Interleukin-6, Unconjugated Estriol, Pregnancy-associated plasma protein, Hemoglobin A1C (HbA1C), and sex hormone binding globulin have been examined in diagnosis of GDM [5,6].

During pregnancy, fat metabolism undergoes physiological changes that increase the production of lipid profiles [27]. Increased estrogen levels and insulin resistance in pregnant women can increase the production of lipids in the liver [28]. These changes in fat metabolism indicate a physiological adaptation in the body of pregnant women that shifts the priority of lipid metabolism over glucose metabolism, and lipids are used as a source of energy for pregnant women so that they can preserve glucose for growth and development of fetal development. Lipids also make it possible to produce embryonic cell membranes, bile acids, and steroid hormones [27]. In early pregnancy, fat accumulation occurs due to increased synthesis of lipids and blood lipids, which increase the level of free fatty acids, especially triglycerides in the blood. On the other hand, increased free fatty acids in the blood can cause insulin resistance [29]. Also, abnormal lipid profile changes are seen in patients with type 2 diabetes [30], so that increasing TG levels above 250 mg/dL and lowering HDL-C levels below 35 mg/dL are considered as a risk factor for type 2 diabetes [31]. Insulin resistance is one of the leading causes of GDM and type 2 diabetes [32]. According to changes in normal pregnancy, insulin resistance occurs due to decreased glucose uptake and increased insulin secretion, and mainly GDM occurs in women whose pancreas does not function sufficiently to compensate for the insulin resistance caused by pregnancy [33]. Also, progesterone plays a role in a way to reset the lipostat in the hypothalamus, leading to increase in the lipids during second trimester of pregnancy [34].

Results similar to present study were observed in a meta-analysis study conducted by Ryckman et al. (2015). TG levels were increased in women with GDM than in women without GDM (95% CI: 25.4–36.4). This finding was consistent in the 1st, 2nd, and 3rd trimesters of pregnancy. HDL-C levels were significantly decreased in women with GDM than in women without GDM in the 2nd (95% CI: −6.2 to −3.1) and 3rd (95% CI: −6.5 to − 1.7) trimesters of pregnancy. No significant difference was shown in TC or LDL-C levels between women with GDM and those without GDM [35].

The present study showed that TG, VLDL-C, and TG/HDL-C ratio were significantly higher in women with gestational diabetes in each trimester of pregnancy than in normal women. HDL in the 1st and 2nd trimesters of pregnancy was lower than the normal group, and TC in the 1st and 2nd trimesters of pregnancy was significantly different in the group of women with GDM and healthy women. But Mankuta et al. observed that TC, LDL-C, and TG decrease in 1st trimesters and increase during 2nd and 3rd trimester. HDL-C levels had no change significantly in the 1st trimester, although it elevated in 2nd trimester and decreased in 3rd trimester [36]. But in other studies it was reported that fat storage increases in the 2nd trimester of pregnancy and causes elevated TG concentration [37].

Correa et al. (2019) evaluated maternal biomarkers in the 1st trimester of pregnancy for early detection of GDM. They showed that there was a significant association between TG, TC, and LDL levels in the 1st trimester of pregnancy with GDM. In this study, lipid profile changes occurred during glycemic normal state and glycosylated hemoglobin [26]. In addition, Layton et al. (2019) conducted a study to determine the lipid profile in women with different sub-groups of GDM. The results of this study showed that there is a significant relationship between TG and GDM. In this study, GDM was grouped into three subgroups, GDM-sensitivity, GDM-secretion, and GDM-mixed, based on measurement of insulin sensitivity and insulin secretion, and there was significant relationship between TG and GDM-sensitivity sub-group compared to the other two groups [38]. In addition, Bukowiecka-Matusiak et al. conducted a study to examine changes in lipid profiles in the membranes of red blood cells in pregnant women with diagnosed GDM. The results showed that TG and TC levels in the group with GDM were significantly higher than that in the group of women with non-GDM [39]. Anjum et al. (2019) investigated the association between HbA1C and lipid profiles with GDM in Saudi Arabian women. The results of this study did not find a significant correlation in terms of TG level between the group with GDM and the non-diabetic group [28]. Besides, the results of Aydemir et al.’s study aimed at examining serum lipoprotein particle levels and its relationship with metabolic status of gestational glucose showed that TG levels were not significantly associated in the two groups of GDM and control group [40]. The reason for the difference in the results of these studies can be considered as not confining the effect of confounding factors on GDM and lipid profiles. On the other hand, these studies measured the levels of lipid profiles using different kits and methods and also different criteria were used for measuring GDM.

Although every attempt to conduct a flawless study was made, this study had some limitations. The authors desired to report age-specific pooled SMD of lipid profile but because most studies did not report age estimate, the authors could not perform the calculations. However, the study had some strong points, as well. For example, it was the first study that reported the overall pooled SMD for lipid profile separated by trimester. In addition, a high number of studies were retrieved in the extensive search and finally 33 studies with a total sample size of 23,792 were analyzed, which provides a sufficient statistical power. Also, we had done unification of units in order to be able to pool the lipid profile. Use of complicate statistical model for unification of SMD and use of fill and trim method for adjustment of publication bias were the strong points of the present study. The other limitations include insufficient studies during the 1st trimester of pregnancy, failure to measure the predictive power of all, studies not examining mothers before pregnancy and during the first trimester of pregnancy in terms of lipid profiles as well as not examining factors such as lifestyle, diet, or other factors involved in increasing the profile of lipids in some studies make it difficult to decide whether to generalize the results.

## 14 Conclusion

Elevated levels of TG in pregnancy occur significantly more in women with GDM than in healthy pregnant women. Higher levels of TC, LDL, VLDL, and TG/HDL ratio and lower level of HDL were exhibited in GDM group. Therefore, TG and TG/HDL ratio can be considered as a possible risk factor and reliable marker in the diagnosis of GDM. Although more research is needed in this area.
